# Results on Three Predictions for July 2012 Federal Elections in Mexico Based on Past Regularities

**DOI:** 10.1371/journal.pone.0082584

**Published:** 2013-12-30

**Authors:** H. Hernández-Saldaña

**Affiliations:** Departamento de Ciencias Básicas, Universidad Autónoma Metropolitana at Azcapotzalco, México, D.F., Mexico; Cinvestav-Merida, Mexico

## Abstract

The Presidential Election in Mexico of July 2012 has been the third time that PREP, Previous Electoral Results Program works. PREP gives voting outcomes based in electoral certificates of each polling station that arrive to capture centers. In previous ones, some statistical regularities had been observed, three of them were selected to make predictions and were published in arXiv:1207.0078 [physics.soc-ph]. Using the database made public in July 2012, two of the predictions were completely fulfilled, while, the third one was measured and confirmed using the database obtained upon request to the electoral authorities. The first two predictions confirmed by actual measures are: (ii) The Partido Revolucionario Institucional, PRI, is a sprinter and has a better performance in polling stations arriving late to capture centers during the process. (iii) Distribution of vote of this party is well described by a smooth function named a Daisy model. A Gamma distribution, but compatible with a Daisy model, fits the distribution as well. The third prediction confirms that *errare humanum est*, since the error distributions of all the self-consistency variables appeared as a central power law with lateral lobes as in 2000 and 2006 electoral processes. The three measured regularities appeared no matter the political environment.

## Introduction

Even when the study and modeling of electoral statistics is an area of traditional interest for Political Economy and, in general, Political Sciences, the availability of databases in the last two decades made electoral systems an area amenable to study for physicists and mathematicians. A wide variety of theoretical models with this point of view exist (see for instance [1] and references therein) and in the last decade the number of studies of actual (empirical) data is growing [2]–[14]. The findings of statistical regularities through several countries and years, encouraged to non political scientist to make guesses or predictions on the results of future elections. Such forecasting stimulates and requires of theoretical frameworks in order to explain the regularities found in the “experimental” data. Notice that these approaches are far from those made by traditional political scientists.

Between the predictions we remark are those of Borghesi [15] which have been verified [16] in relation to electoral turnout. Here we present the results for three predictions made before the July 2012 Mexican electoral process and made public in [17] previously to the election. As we shall see, two of them were fulfilled with the original dataset provided by the official channels and the third one was incomplete due to the change in the official data presentation, which forbade the publication of the self-consistency data while the certificates were processed.

## Results and Discussion

### Data and Observables

The analysis is performed on the dataset provided by the electoral authorities through the *Programa de Resultados Electorales Previos*, PREP or Previous Electoral Results Program, during the election day and the next one. On how this program is implemented see the official electoral authorities web page [18], [19] or reference [17]. On the peculiarities of the Mexican electoral processes see, for instance, [20]. Upon request, the electoral authorities gave access to the self-consistency additional data and the corresponding analysis is presented here [21].

The database for the whole process contains the fields recording polling stations IDs, number of votes for each political party/candidate, time of arrival and a set of control fields that are summarized in Table 1. We consider 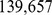 valid records, from a total of 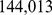, in the dataset provided by the electoral authorities [21]. For 2000 and 2006 elections we used the dataset from references [18], [19].

**Table 1:** Self consistency fields and errors considered in the PREP database of July 2000, 2006 and 2012.

E_1_	B. received	Br - (Bs + V)
	− (B. not used + Number of voters)	
E_2_	B. received	Br - (Bs + Bd)
	− (B. not used + B. deposited)	
E_3_	B. received	Br - (Bs+Σ*_i_* V*_i_*)
	− (B. not used + Votes for each party)	
E_4_	Number of voters	V - Bd
	− B. deposited	
E_5_	Number of voters	V - Σ*_i_* V*_i_*
	− Votes for each party	
E_6_	B. deposited	Bd - Σ*_i_* V*_i_*
	− Votes for each party	

We abbreviated Ballots with B.. The variable V*i* stands for the number of votes obtained for each party/candidate.

For the first analysis we use the distribution (not a normalized histogram) of the variables E*_i_* which are built up from the values of the control fields described in Table 1; there, the six independent combinations available are considered. The variables are built up in order to see the lack or excess of votes in the records, for instance the total number of voters must coincide with the number of deposited ballots in the urn (E_4_). In the ideal case all the distributions must be Dirac's delta functions, i.e., the distribution is zero everywhere except at the origin. So, these distributions are, in fact, the error distributions.

For the next study, we consider the percentage of votes for a party (PRI, in the current paper) at a certain time *t* or at a certain percentage of processed votes certificates. For the third subject instead of the percentage of votes obtained we consider the distribution of votes: it is made by the histogram of the number of polling stations with certain amount of votes, properly scaled and normalized in order to have a distribution normalized to unity with unit mean as well. Since we wish to compare with a probability distribution the amount of votes is “unfolded” or “deconvoluted” by using the average number of votes, which properly scales the variable. The resulting histogram must be normalized to area one. (In reference [22] there is an explanation about this procedure, but is standard in data treatment.) For simplicity we focus only on results from the presidential election. In the next paragraphs we shall explain the proposals made in [17] based in our previous analysis and how they are accomplished for the 2012 Mexican election. We shall use the word “prediction” to describe the statistical regularity which has the same character in 2012 as in previous elections.

### Prediction i) Errors could be epidemic in contemporary Mexican elections

Control fields or self-consistency records in electoral data are an important measure in order to test and understand the sources of error. The distribution of such records were presented in the first version of [23] for the July 2006 federal elections for presidential and for both chambers and, for the presidential election of 2000 in an ulterior version. The regularities appeared for the error distributions described in Table 1, allowed me to formulate the following forecast for the 2012 case:

*Error distributions in self consistency tests of PREP's dataset will be described globally by a power law at the center and two asymmetric lobes at each side.*

**Figure 1:**
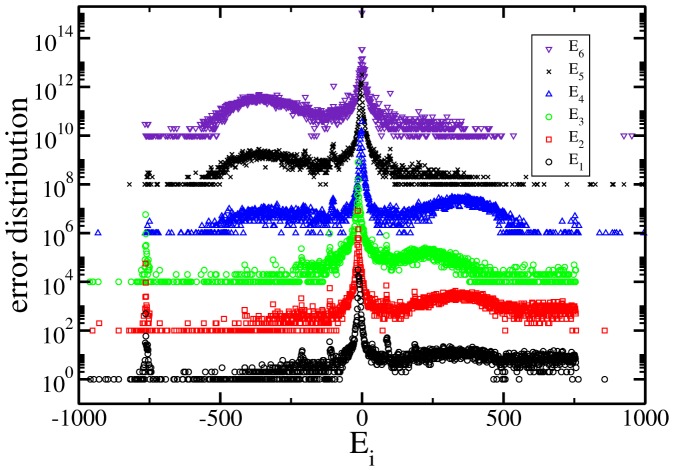
Error distributions for the variables described in Table 1 (from bottom to top) for the presidential election on July 2012 in Mexico. The distribution are plotted in log-linear scale. We scale by a factor of 100 each time. Notice that they are characterized by a power law at the center and asymmetric lobes at each side. In Figure 4 the power laws are exhibited.

**Figure 2:**
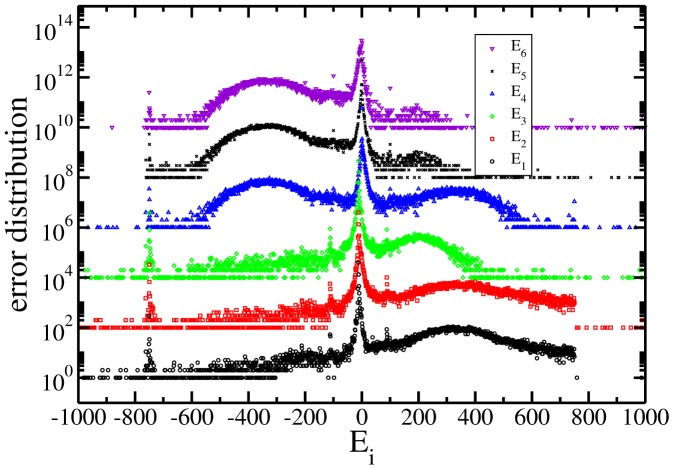
Same as previous for the presidential election of July 2000 in Mexico. See text for details.

**Figure 3:**
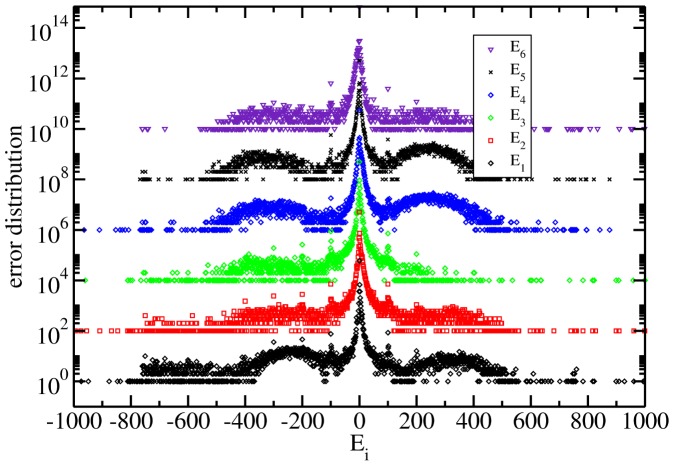
Same as previous for the Mexican presidential election of July 2006. See text for details.

The six independent distributions of *E_i_* are shown in Fig. 1 for the presidential process of July 2012. As can be seen there, the whole behaviour is similar to that in the distributions calculated for the 2000 (see Fig. 2) and 2006 (Fig. 3) processes. As explained below they are the histograms of the number of cabins that have values of error equal to 0,1,2,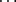. For instance, to built up the distribution of E_4_ we evaluate the difference between the number of voters, V(*k*), reported in the urn *k* and the number of ballots deposited, Bd(*k*), i.e., E_4_(*k*) = V(*k*)− Bd(*k*). This difference could be 0,1,2,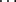, or even 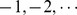 if the numbers of ballots deposited are larger than the number of voters. With these values we built up the histogram of how many polling stations present a difference of 0,1,2,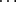 or 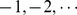. In the ideal case, the total number of cabins, *N*, must present a difference of zero, or E_4_(*k*) = 0 for all *k*. In such a case the histogram is a bin at zero with a high of *N*, all the other bins have high zero. In common examples of this kind of histograms a Gaussian appears, but not in Mexican elections. Certainly, these distributions can be interpreted as appearance and missing of votes, but in fact they are a measure on how we count the electoral results. The errors can be intentional cheating or counting mistakes.

For the present case some changes appeared in the labels of the dataset, for instance, the “Total number of Ballots deposited”, Bd, are now “Total number of extracted ballots from the urn”; the sum of all the votes for the different political parties and candidates is now made directly by the IFE's computers. In the last case, we test the value calculated by the computers and the sum on the records with no main differences. It is important to notice that the final electoral results are presented and accounted after all the parties reached an agreement on the results in each polling station.

**Figure 4:**
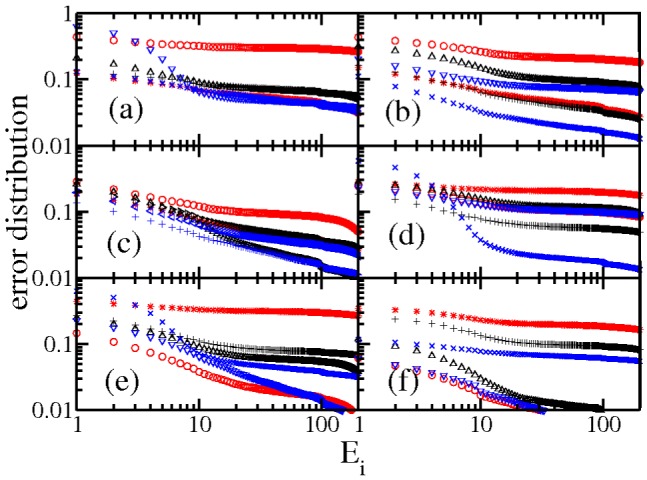
Normalized error distribution by wing in log-log scale, organized as in Table 1. The labels correspond with (a) for E_1_ up to (f) for E_6_. In red the 2000 case, in black the 2006 and in blue the 2012, in geometrical symbols the right wing and in cross, plus and star the left wings. Notice that almost all the cases are straight lines in this scale, i.e. the decay is like 1/E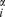.

In the figures from 1 to 3 the distributions are scaled by a factor of 100 each time and in a log-linear graph in order to appreciate all of them in a single figure. In all the cases the central part has a power law decay and two asymmetrical lobes. In order to appreciate the power law decay at the center in Fig. 4, the integrated distribution is shown in log-log scale for all the cases and for both wings, left(LW) and right(RW). We reflected the left wing as a way to use log scale. In the figure both wings were normalized to unity. There are three regimes, one from 1 to 10, other from 10 to 100 and the third one characterized by the lobes (not shown). Almost all the distribution wings at the center are characterized by a power law but it does not look with a universal character.

As another characteristic, large peaks appear in all the graphs in Figs. 1 to 3. The reasons for this behaviour is unclear but it looks as a general feature that deserves a wide and detailed study [24].

### Prediction ii) The Partido Revolucionario Institucional (PRI) is a sprinter

Even when the behaviour presented here for the presidential candidates of the Partido Revolucionario Institucional appeared in election for the both chambers we shall concentrate in the presidential case. A graph of the percentage of votes for each party/candidate against the percentage of computed polling stations had been presented in voters outcome reports for federal elections in 2000 and 2006. In reference [23], version 3, both elections are reported, in Figure 1 and 2 for 2006 and, at the end, the corresponding for 2000. In all the analyzed cases, the PRI showed a change in the percentage of votes' slope. Close to the 70% of computed polling stations an increase in the percentage of votes is evident. No matter that in both elections this party did not obtain the largest amount of votes, it appears ruling in polling stations arriving at the end of the counting process. It is a well known fact, due to historical reasons, that PRI receives a lot of votes in geographical regions with a high marginalization index (see for instance [25]), such regions are expected to have a slow electoral data processing and transmission to capture centers. This might explain why PRI is a sprinter. In [17] the statement for the 2012 election was:

*In the graph of percentage of vote against percentage of processed certificates the PRI will change its rate of growth around the time when 70% of the computed certificates arrive. i.e. this political party has a good final sprint.*

**Figure 5:**
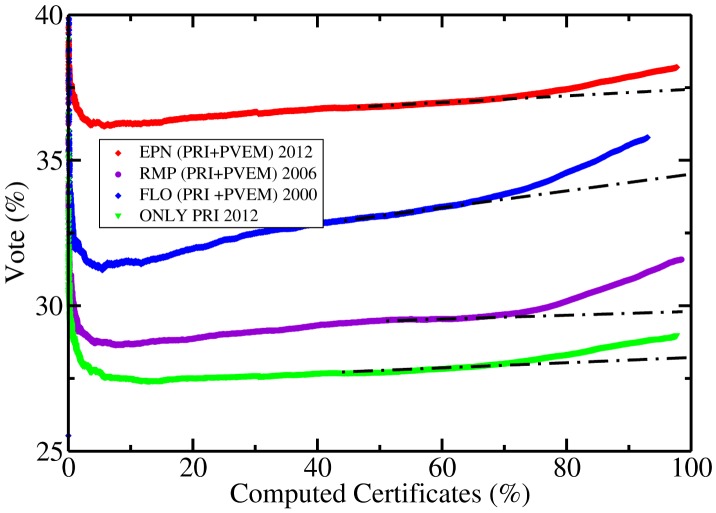
Percentage of votes obtained by the Partido Revolucionario Institucional, PRI, presidential candidate in the federal elections of 2012 (upper red line), 2006 (second from the bottom in violet line), 2000 (third from the bottom in blue line) and percentage of votes for PRI alone in July 2012 process (first line in green). Straight dotted lines are included to guide the eye. In 2000 and 2006 only the vote for coalitions was admitted. For the 2012 process see explanation in the text. Notice that *in all* the cases the graphs present a change in slope around 70% of computed certificates.

In order to test it, we report, in Figure 5, the percentage of vote obtained by PRI against the percentage of computed certificates of the polling stations. We report the presidential candidates in 2012 (EPN, Enrique Peña Nieto), 2006, (RMP, Roberto Madrazo Pintado) and, 2000 (FLO, Francisco Labastida Ochoa). For the July 2012 election the rules changed, candidates in coalitions appeared in the ballot in two ways, in coalition and as candidate of one party. So, we can differentiate the votes for PRI only, from those obtained by the options PRI+PVEM and PVEM alone. Hence, we present the case for PRI alone as well. As can be observed in the Figure 5, for all of these cases the PRI changes its growth slope, increasing, in a noticeable way, the percentage of votes. No matter if we analyze PRI alone or the coalition. The change in slope is different in all the cases. The small party in a coalition presents a typical small party behaviour (not shown). Hence, *from* *Figure 5* *it is clear that statement ii) has been verified.*

Some small details about Figure 5. All the polling stations certificates were considered in the figure, hence it has small fluctuations that are not appreciable due to the plotting character size. The present figure was processed in order to keep the file size small. The PREP record ends at a certain hour, usually 24–26 hours after the beginning of capture and does not include 100% of the polling stations. So the end of records is different for each process.

**Figure 6:**
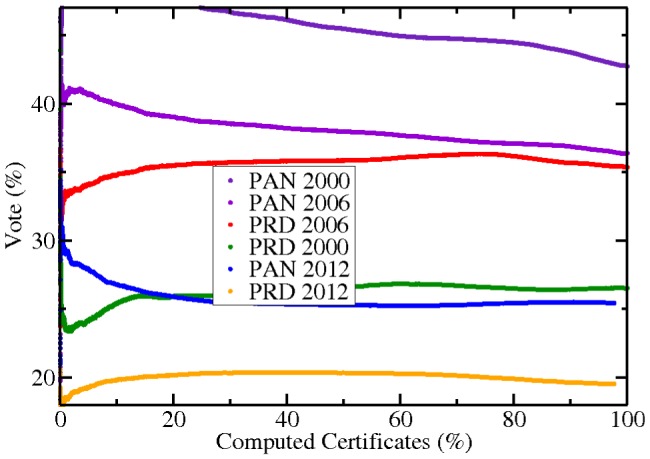
Percentage of votes against the percentage of computed certificates for the three elections and for the other two candidates. The order of the graph is the same as the labels in the inset. Notice that all the curves decay in some degree after the 70% of the certificates have been computed.

For completeness, in Fig. 6 we show the results for the two other main participants during the last elections. As can be seen, the slope is negative for all the cases beyond the 70% of computed certificates, except for PAN 2012. Notice that PAN 2000 (at top of the figure, in violet) decreases its percentage of votes during the vast majority of the computation. This is consistent the growing rate of PRI during the same year (blue dots in Fig. 5). See as well that both the PAN and the PRD evolution falls down for the the 2006 case after the 70% of counted certificates.

### Prediction iii) The PRI has a smooth vote distribution

Beyond the important discussion about universal features in vote distribution in world wide elections, a corporate political party has been extremely regular: the Partido Revolucionario Institucional (PRI). In all the previously performed analysis, [7], [22], [23], [26] its vote distribution is a smooth function. In reference [7], the smooth behaviour of this party in federal elections 2000, 2003 and 2006 using the definitive dataset of Count by District was reported. A similar behaviour has been observed in the 1997 and the 1994 elections by the author but the results remain unpublished. Two main characteristics appear in this vote distribution: 1) The number of polling stations where the PRI obtained few votes is small, and the probability to obtain less than 10 votes in each urn is practically zero for all the presidential elections. 2) The distribution is peaked one, that is, the party has a mechanism in order to obtain more or less the same amount of votes. The value of the mean varies from election to election but the distribution type is remarkable constant. The other two major parties do not present such a regularity and, they seem to be the sum of two different distributions. Meanwhile, the small parties, independent candidates and null votes follow a shifted power law. In this way the consistency of the PRI through the years is noticeable and it seems as the fingerprint of this corporate party.

The distribution of votes we consider is the histogram of the number of polling stations with a certain amount of votes, properly scaled and normalized in order to have a normalized to unity distribution with a unit mean as well. In order to do the comparison with a probability distribution the amount of votes is ““unfolded” or “deconvoluted” by using the average of the number of votes, which scale properly the variable. The resulting histogram must be normalized to area one.

After this process, fitting a model is possible. Daisy functions [27], of different ranks, were tested with success for the 2000, 2003 and 2006 electoral processes for president and for both chambers. The only free parameter in this model is the rank, *r*, and it is written as:
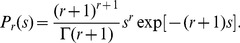
(1)With *r* an integer and 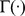 the Gamma function.

However, this distribution is a particular case of a more general distribution named the Gamma distribution. It is characterized by two real free parameters, *α* and *θ* [28], [29] and written as:
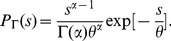
(2)When 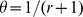 and 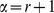 we recover Eq. (1).

In this term, the third guess was presented as:

*The distribution of votes for PRI, in presidential and both chambers elections, fit smooth distributions, in general a Gamma distribution or by Daisy models.*

**Figure 7:**
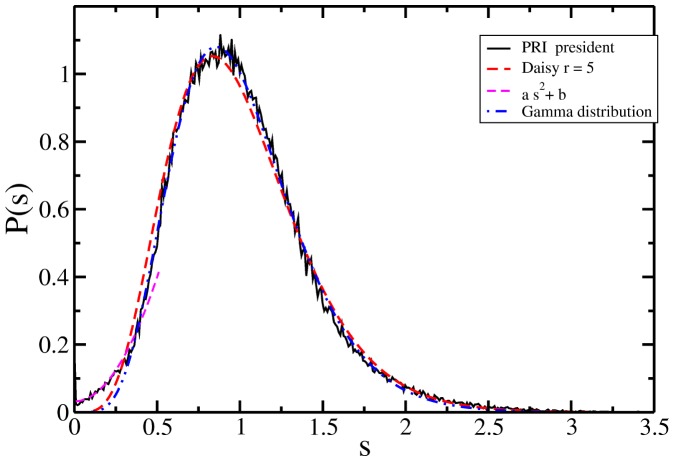
Comparison of the July 2012 PRI distribution of vote for president (black line) with the two models. In broken red line a Daisy model of rank 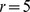, equation (1), in blue dot-line the Gamma distribution, equation (2), with the best fit. And in magenta broken line a quadratic polynomial which fits the distribution's beginning.

The result for the 2012 case is presented in Figure 7 and corresponds to the presidential case for the votes for PRI alone. We left the other cases for a future work. There, the normalized histogram is presented in a black line. It is noticeable that the beginning of the distribution is not compatible with the fast decay at the tail and presents an abrupt change in slope (not shown). Such behaviour certainly can be analyzed with the Gamma distribution, but we keep the analysis apart since this kind of change in the slope has been reported for the 2003 intermediate elections. There, the behaviour corresponds to a different dynamic. The beginning of the distribution in Figure 7 is fitted by a quadratic polynomial with no linear term.

For the distribution remaining part we test our two models and we test a log-normal distribution as well. In broken red line appears a Daisy model of rank 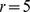 which follows the curve nicely.

In order to test how good the Daisy model is, we contrast it with the Gamma distribution with two free parameters, equation (2). The fit was obtained for different starting points, since the change in slope is at 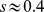. For fittings starting beyond this point the results are around 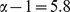 and 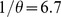. All the results are compatible with a Daisy model, since the relation between *α* and *θ* remains as 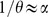 and both parameters are close to an integer.

**Figure 8:**
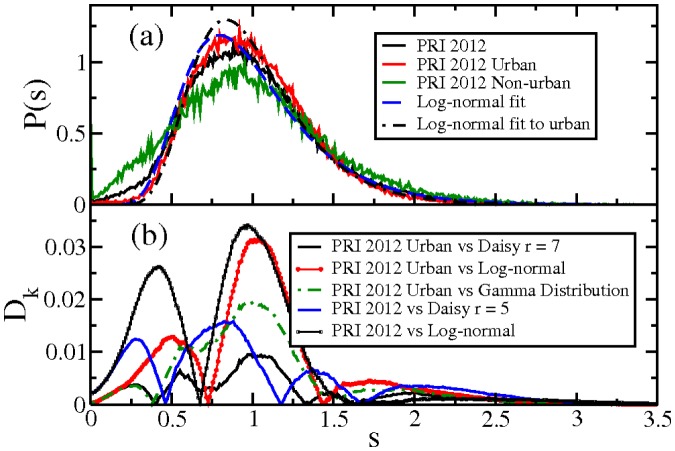
Kolmogorov-Smirnov test for the July 2012 PRI distribution of vote. (a) Distribution of vote for the PRI during 2012, we separate the urban and non-urban voters. (b) Distances between the cumulative distributions indicated and the experimental distribution given by the PRI See text for explanation.

Furthermore, in order to avoid the behavior at the beginning of the distribution, we shall concentrate on the urban vote. In Fig. 8(a) we show the corresponding distributions for urban and non-urban vote. It is clear that non-urban vote contributes greatly to the distribution beginning, that is, in non-urban scenarios there was, in this election, more polling stations with a relatively small amount of votes for PRI. The distribution for urban voters is thinner. A Daisy model of rank 7 fits well the distribution as we shall explain later. In the same Figure the best fit to the data with a log-normal distribution is shown. Such a distribution is written as
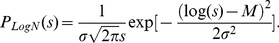
(3)where *M* and *σ* are real parameters. The mean is given for 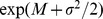 and, in our case we set it to unity. Hence, the distribution depends only of *σ*. This distribution appears in several electoral analysis [2], [10].

We use a Kolmogorov-Smirnov test to analyze the statistical significance of all the models. For this test we require to calculate the maximum difference between the cumulative distribution of the experimental data 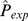 and the model:
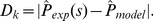
(4)Such a difference is shown in Fig. 8(b) for the Daisy models and log-normal distributions in both cases, the urban and the whole vote distribution. The critical value 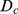 in order to accept or not the test is proportional to 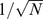, for large the number of points *N* considered. In our case 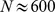, i.e., around 0.04. From the figure is clear that there is not a value of 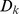 larger than 0.04. As a consequence, we can not reject the models. But the Daisy model have smaller differences and we can not reject the *proposal iii) that PRI has a smooth vote distribution described by Daisy models.*

A detailed analysis of the exact values of the parameters is irrelevant at this moment since we do not have a theoretical model that explains this smooth behaviour. There are efforts in this way [22], [26]. Additionally, the results can be a mixture of dynamics since the histogram is built up using the complete database and does not consider differences in district or state, or if the state has been ruled by PRI for a long time. It is important to know that in several Mexican states PRI rules since 1929. The analysis of PRI's distribution of votes performed by state and district is in progress.

### Conclusions

Any scientific work must provide predictions, even when they could be based only on empirical observations. To have a valid theoretical framework for the regularities is a much more satisfactory result, but social systems are not well understood and this opens wide opportunities for research and for new multidisciplinary approaches.

In this paper I offer evidence that Mexican elections, as many others in several countries and years, present regularities. The first fulfilled statement is of a general nature and it says that errors are always present, due to honest mistakes or to intentional cheating. Here we show that for the third time in a row, Mexican elections show characteristic distributions of error in the self-consistency records. The reason for this behavior is unclear and deserves a separate analysis [24] since they appeared in datasets that correspond to contrasting political environments: 1) lack of suspicion of fraud in presidential election (July 2000) with the defeat of the long time ruling party, 2) large suspicion of fraud (July 2006) and, 3) comeback to power of the ancient political party. Hence, this kind of behavior appeared in all of them. Certainly, local fraud by *all political parties* in Mexico is well documented, and we hope that some of the common practices appear under a much more detailed analysis.

The second successful regularity is a result of history and geography: PRI has a well established promotional system that has ruled for many years. So, no magic intelligence needs to be invoked in order to explain this time domain behaviour. Wider studies could confirm this. The third accomplished regularity is a much more delicate question. The appearance of probability distributions in a process is, in general, evidence of some sort of general principle behind it. Such is the case of Gaussian distributions or power laws. The appearance of Daisy models in all of the PRI electoral process could open a door to understand corporative practices of parties around the world. It is important to remark that during the 2006 election the PRI candidate was the *third* option during the whole campaign. So, the votes it received was due to the very core of the party, i.e. a corporate party who ask for quotes of votes. For the 2012 case, allegations of buying of votes were makde, however this is a common practice through the history of this party as has been reported in the political sciences literature. The conclusion is that the PRI has very consistent systems to obtain votes and the smoothness of its distribution of vote is characteristic.

## Discussion

### Materials and Methods

All the used datasets are public via the web page of IFE [18]. Some dataset are available upon request through the IFE authorities [21] or the author's e-mail. The files in text format contain empty fields or comments, some of them are explicit in the annexed documentation. For simplicity I did not consider any polling station recorded with an empty field. The analysis of the error distribution of 2006 PREP was performed again with the data base of *accepted* polling station certificates. All the data treatment was made in Fortran 77 and the source code is available from the author.
